# Dynamics of the *O. felineus* Infestation Intensity and Egg Production in Carcinogenesis and Partial Hepatectomy in the Setting of Superinvasive Opisthorchiasis

**DOI:** 10.1155/2019/8079368

**Published:** 2019-07-24

**Authors:** Vitaly G. Bychkov, Ludmila F. Kalyonova, Elena D. Khadieva, Semen D. Lazarev, Ilgiz R. Lukmanov, Vladimir A. Shidin, Evgeny N. Morozov

**Affiliations:** ^1^Tyumen State Medical University, Tyumen, Russia; ^2^Tyumen Scientific Centre, Tyumen, Russia; ^3^Khanty-Mansiysk State Medical Academy, Khanty-Mansiysk, Russia; ^4^Sechenov University, Moscow, Russia; ^5^Russian Medical Academy of Continuing Professional Education, Moscow, Russia

## Abstract

Clinical and experimental studies have shown that opisthorchii tend to evade tumour growth foci to colonize more distant areas of the liver. When modelling tumours with various carcinogens in the setting of superinvasive opisthorchiasis, the intensity of invasion is reduced both before the formation of neoplasms (>120 days) and after the development of tumours of various histogeneses (liver, pancreas, and stomach) (>240 days). Egg production was observed to increase with the decrease in the number of parasites in the liver. The smallest changes in the infestation intensity indicators and egg production were observed in the experimental stomach tumours (*p* > 0.05). A partial hepatectomy in the setting of opisthorchiasis did not affect the number of parasites in the ecological niche (liver) or the production of eggs by the helminth. With the deterioration of the vegetation state, parasite clumps of opisthorchii increase egg production under the conditions of distress.

## 1. Introduction

Human opisthorchiasis is a parasitic disease discovered by Professor K.N. Vinogradov in 1891 in the city of Tomsk (Eastern Siberia), the causative agent of the disease—the trematode Opisthorchis felineus, Rivolta, 1884. The literature describes the changes in the internal organs with this helminthiasis [1]: features of tissue regeneration and development of atherosclerosis [2, 3]; pathologies of the liver and pancreas, where parasites vegetate [4, 5]; and morphological changes in the lungs with SO [6]. Of particular importance is the study of the cardiovascular system in opisthorchiasis invasion with repeated infections [7, 8]. Currently, the role of superinvasive opisthorchiasis in the development of liver malignant neoplasms, which in the hyperendemic foci of helminthiasis are significantly more frequent than in regions without this invasion, has been identified: CO is a strong promoter in the carcinogenesis of not only the liver but also the pancreas, stomach, and other organs [9, 10]. The aim of the current study is to identify patterns of the intensity of the invasion and egg production of Opisthorchis felineus in the carcinogenesis of various organs and partial hepatectomy in the setting of superinvasive opisthorchiasis.

## 2. Materials and Methods

According to the data from postmortem examinations, loci of *O. felineus* vegetation were found in the liver in superinvasion (*n* = 88), liver cancer (*n* = 42), pancreas (*n* = 18), and gastrointestinal stomach tumours (GIST) (*n* = 29) in the setting of SO. In this study, tumours were simulated by chemical carcinogens: N-DMNA, N-DENA, N-MNNG, NDMM, NDOP, and DMBA (N-dimethylnitrosamine, N-diethylnitrosamine, and N-diethyl-itronitroso-guanidine, 2,6-nitrosodimethyl-morpholine, 2,2-nitrozodioxypropylalanine, etc.). Repeated infestations occurred on days 16, 32, 60, 120, and 240 after the primary infection. Methodological aspects of tumour modeling in the setting of SO were published previously [11].

Partial hepatectomy in Syrian hamsters (*n* = 48) was performed by removing a liver lobe, which accounted for 17.3-17.7% of the organ weight. The opisthorchiasis and SO model was developed in mature Syrian hamsters (*n* = 280) weighing 98.0-110.0 g. The larvae of opisthorchii were prepared by means of artificial gastric juice (pepsin+hydrochloric acid+water) according to the method of Glazkov [12]. The invasive material, *O. felineus* metacercariae, was introduced into the stomach; repeated infestations were performed 16 and 32 days after the primary infection. The animals were etherized (lethal overdose). The reproductive activity of opisthorchii was determined by the native smear, Fülleborn, and Thalemann methods. The infestation intensity was determined via incomplete helminthological dissection of animals according to K.I. Skryabin.

Liver and stomach preparations were stained with Mayer's haematoxylin and eosin according to Van Gieson and with alcian blue and Schiff's reagent according to McManus. The IHC test was performed with antibodies against the CD117, DOGI, and CD34 protein receptors and cytokeratins 7 and 19 according to the manufacturer's recommendations (Leica Biosystems and Spring Biosciences; the ultrastructural study was performed on a JEM-1011 microscope (Japan)).

Animal experiments were carried out in accordance with the principles set forth in the European Convention for the Protection of Vertebrate Animals used for Experimental and Other Scientific Purposes (Strasbourg, 1986), with the rules of laboratory practice of the Russian Federation, and with Order No. 724 of 1984 of the Ministry of Higher Education of the USSR, “Rules for carrying out work with experimental animals,” after obtaining permission from the ethical committee of the FSBEIHE Tyumen State Medical University of the Russian Ministry of Health.

Statistical analyses of the actual data were carried out using the licensed software SPSS Statistics (USA) and Microsoft Excel by MS Office 2016 for Windows; single-factor ANOVA was used. The differences were statistically significant at *p* < 0.05.

## 3. Results and Discussion

A study of the dynamics of the infestation intensity and the reproduction of the population and individuals of *O. felineus* during a single infection showed that after 21 days, opisthorchii of different degrees of maturity were simultaneously detected in the liver and pancreas, and in faeces, helminth eggs with a low reproductive potential were detected (2.0 ± 0.7 eggs per parasite). The most pronounced rise of all of the indicators was observed on days 30-40 after infestation (infestation intensity—40.2, number of eggs per parasite—57.3 ± 12.3). The highest indices were observed on days 48-56 after the start of the experiment (egg production by 1 parasite was 76.0 ± 11.4).

Statistical analysis of the results in the setting of SO showed a significant increase in mature opisthorchii after 48 days, with a maximum reproductive potential (54.5 eggs); as the duration of superinfestation increased, the intensity of the mature opisthorchii increased as well (88.2) but did not double (infection—50 metacercariae, repeated inflows—50 larvae) until the end of the experiment (320 days). The parasite quantities in hamsters with SO are shown in Table 1.

**Figure 1:**
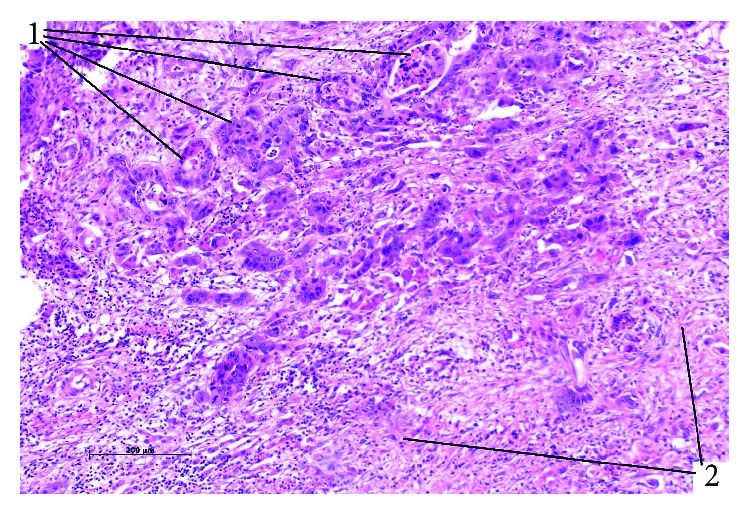
Cholangiocellular tumour with a forming coarse stroma in the setting of superinvasive opisthorchiasis (SO) was visualized with haematoxylin and eosin (HE) staining, magnification 200x (1: tumour glandular complexes; 2: forming coarse stroma).

**Figure 2:**
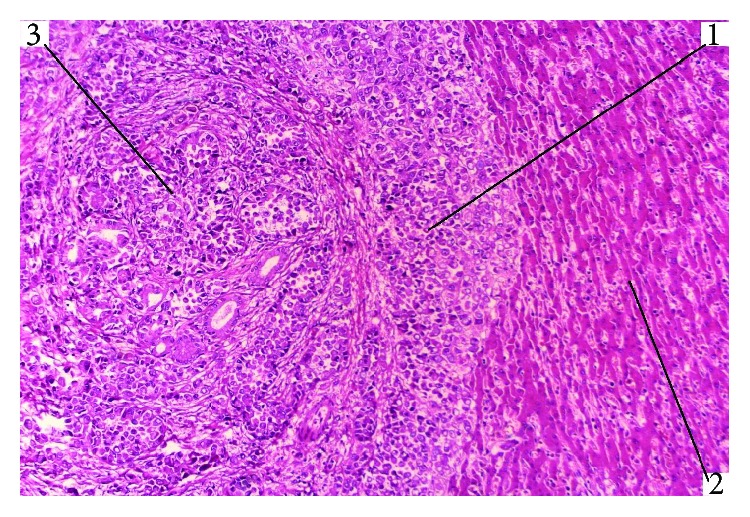
Undifferentiated cancer in the setting of SO. Opposition growth. HE staining, magnification 200x (1: undifferentiated tumour cells; 2: normal liver cells; 3: cholangiocyte adenomatous proliferates).

A pathoanatomical examination of the liver of animals that died from cholangiocarcinoma (CCR) and other histological forms of tumours (Figures 1 and 2) showed the presence of 1 or more nodes; the largest number of tumours was observed in the left lobe of the organ, and intraorgan metastases were noted as foci with a rounded shape and clear edges.

**Figure 3:**
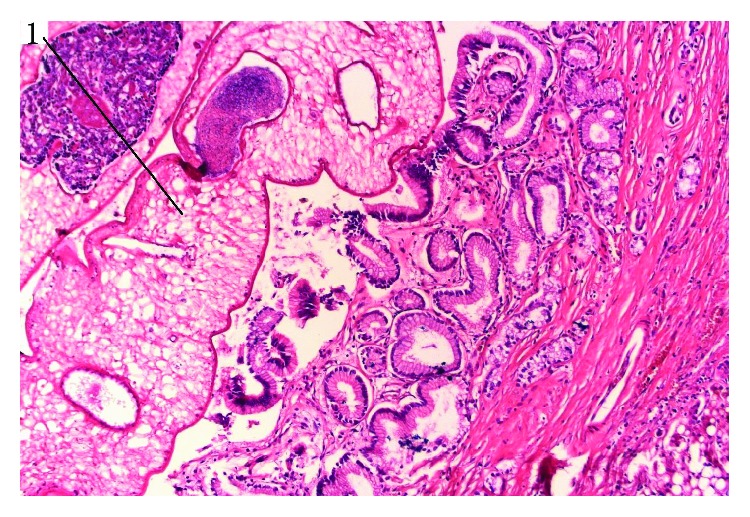
Opisthorchii in a duct located remotely from the tumour (showed by arrow). HE staining, magnification 200x.

**Figure 4:**
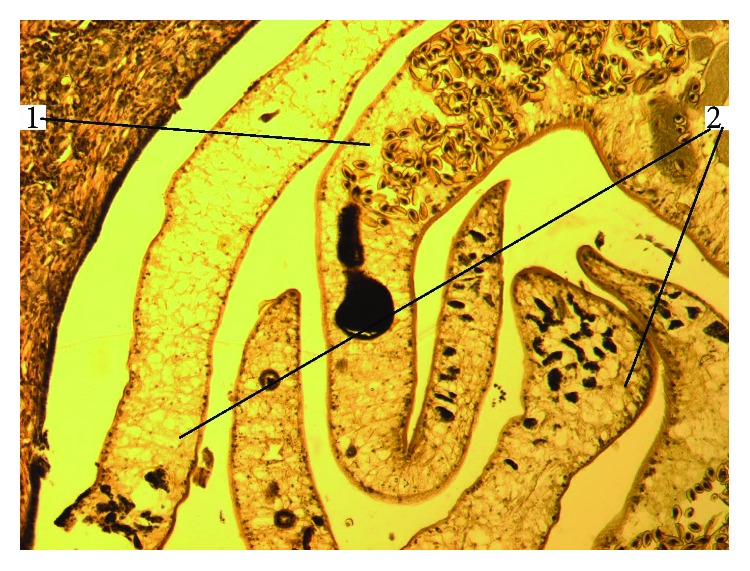
Clumps of opisthorchii in cholangiectasis in liver cancer in the setting of SO. HE staining, magnification 100x (1: mature parasites; 2: immature parasites).

Parasites were not found in the ductal system adjacent to the tumour. Clumps of opisthorchii were found in areas remote from tumour nodes (Figure 3). Observations of CCR with metastases into portal lymph nodes showed bile hypertension syndrome and formed cholangiectasis, where a large number of helminths predominantly grew in the form of small “lumps” consisting of 12-16 individuals. In a larger ectasias, such cooperation consisted of 22 or more parasites (Figure 4). Single, smaller knots of opisthorchii were detected in enlarged bile ducts, mainly in other parts of the liver that were free of tumour growth.

Parasites in tumour-free segments were closely attached to the lining of the duct, occupying the surface along the entire length of the helminth, i.e., they occupied a position that facilitated contact with the largest area of the mucous membrane of the ducts.

**Figure 5:**
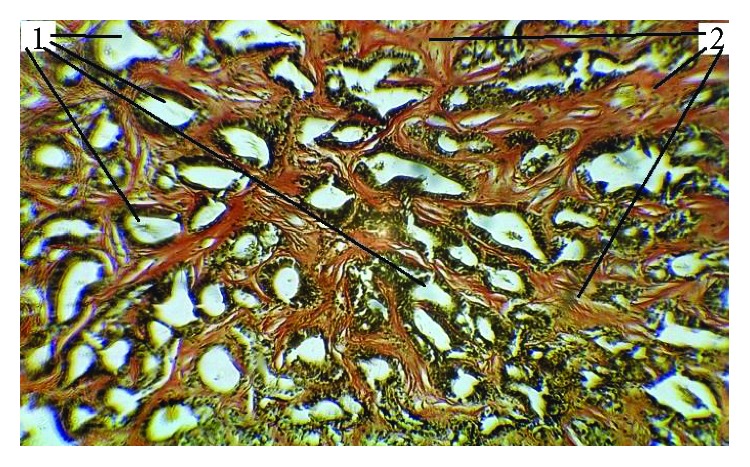
Experimental cholangiocarcinoma with a coarse stroma in the setting of SO. Van Gieson's staining, light microscopy magnification 20x (1: tumour glandular complexes; 2: coarse stroma).

**Figure 6:**
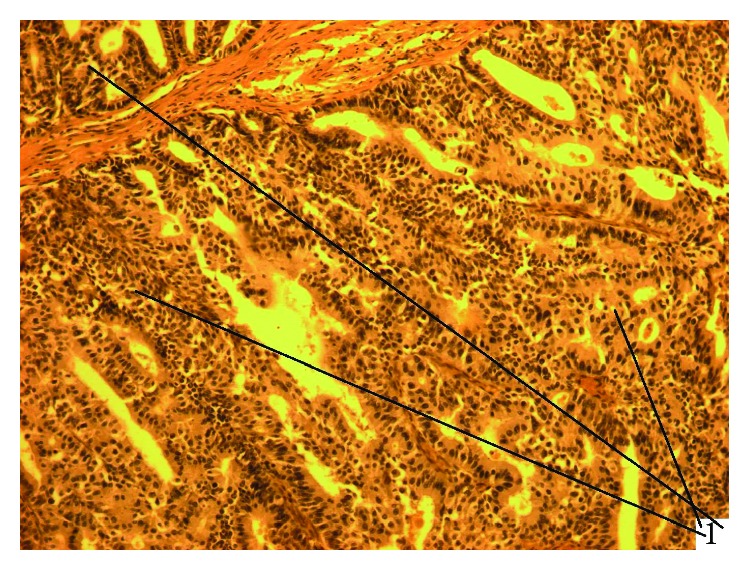
Experimental cholangiocarcinoma with a tender stroma in the setting of SO. HE staining, magnification 200x (1: adenocarcinoma).

**Figure 7:**
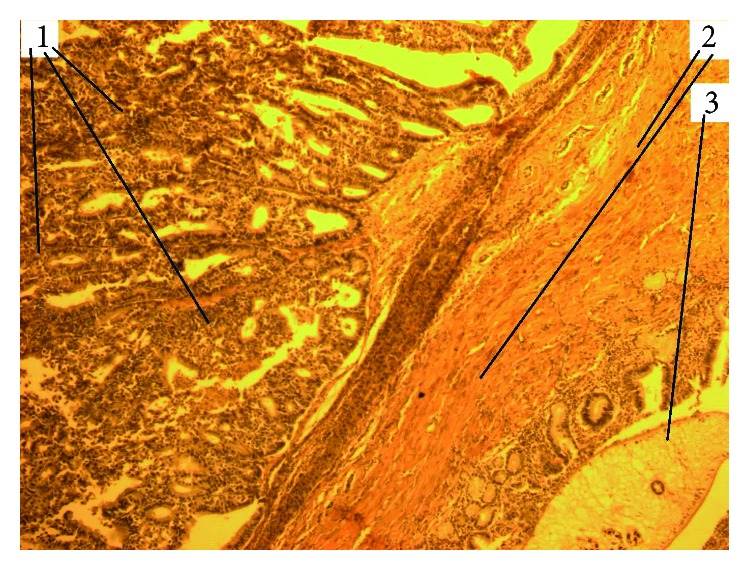
Experimental cholangiocarcinoma with a tender stroma in the setting of SO. HE staining, magnification 200x (1: adenocarcinoma; 2: coarse cholangiectasis wall; 3: opisthorchii in cholangiectasis).

**Figure 8:**
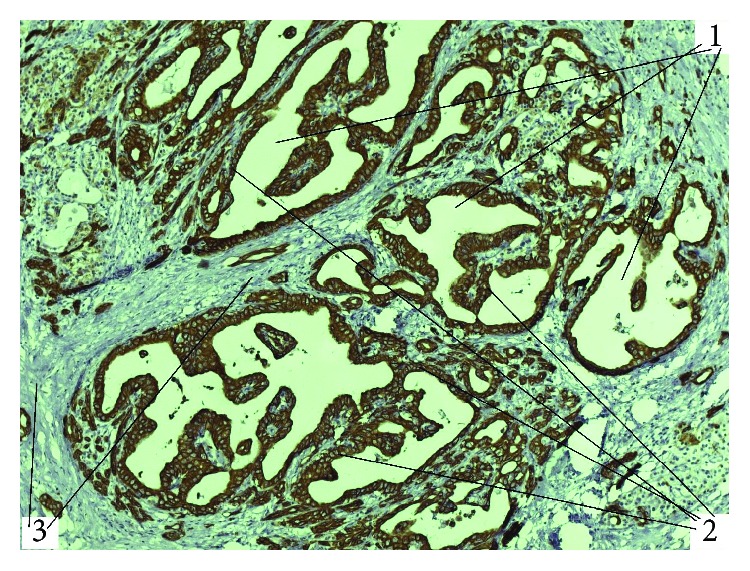
Experimental pancreatic cancer in the setting of SO, IHC reaction with antibodies against cytokeratin-19 receptors, magnification 100x (1: cyst component; 2: papillary component; 3: tumour stroma).

**Figure 9:**
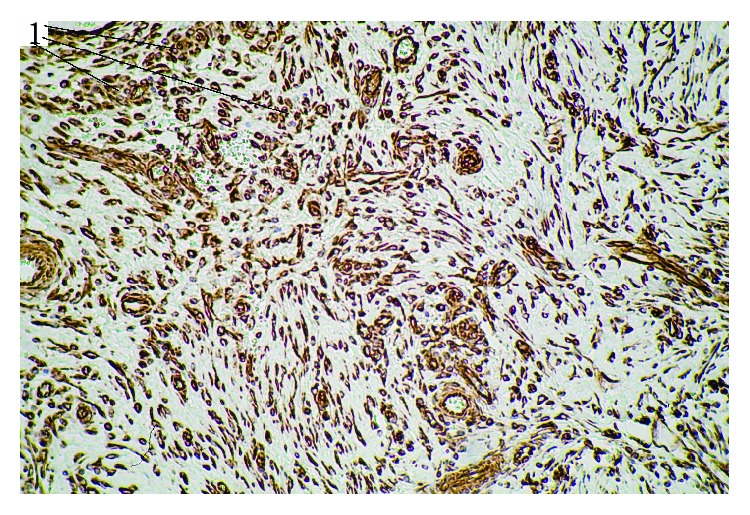
Experimental gastrointestinal stromal tumour (GIST), IHC reaction with antibodies against CD117 receptors, magnification 200x (1: tumour elements from cells of Cajal).

Simulation of tumours in the setting of SO revealed the development of neoplasms in 38.4-76.9% of all cases, and the multiplicity factor varied: 1.46 in the liver (Figures 5–7), 1.32 in the pancreas (Figure 8), 1.27 in the stomach (Figure 9), 1.11 in the mammary gland, and 1.32 in the skin, without tumours; all of the tumours were nonlethal. Tumours with metastases to the lymph nodes were identified in CCR and adenocarcinoma of the pancreas. GISTs and metastases to the liver were also observed. In groups with numerous superinvasions, the numbers of tumour formations in the liver, pancreas, and stomach increased by 16.7-22.3%, and metastases of tumours into lymph nodes and other organs were more often noted. A sharp decrease in the intensity of infestation and an increase in egg production were observed on day 85 of invasion and day 65 of superinvasion (Table 2).

In the experiments for partial hepatectomy in the setting of SO, by day 48, the number of parasites in the liver was 46.3 ± 3.17, the number of eggs in 1 g of faeces was 2673 ± 94, and the egg production index of 1 opisthorchis was 55.7 ± 8.2. The morphological examination showed a uniform distribution of helminths throughout the organ, but the most populated ducts were noted in areas adjacent to the liver stump, where multiple newly formed ducts and vessels were formed.

## 4. Conclusion

The development of tumour processes in the human liver makes opisthorchii move away from the sites of neoplasm localization. The main areas where opisthorchii are found are large ducts and cholangiectases, where helminths create more compact populations, which indicates an insufficient nutraceutical supply.

Model tumours with SO induce a similar effect: initiators (carcinogens) provide a partial anthelmintic effect—a decrease in the intensity of infestation preceding the formation of neoplasms; the further population-level depression might be due to a lack of nutrition for parasites and the disruption of homeostasis.

A decrease in the infestation intensity regardless of the cause (carcinogens, reduction of the nutraceutical substrate) stimulates egg production of the remaining helminths, which is a trend of species preservation. Negative effects on helminths or their death results in an increase in egg production by surviving individuals, which indicates the intraspecies social relations of opisthorchii.

## Figures and Tables

**Table 1:** Dynamics of the infestation intensity and egg production of *O. felineus* in experimental opisthorchiasis (SO).

Time of infestation/superinvasion (days)	Number of opisthorchii in ecological niches	Number of eggs in 1 g of faeces	Number of eggs per parasite
21/6	34.2 ± 3.3	57 ± 28	1.7 ± 0.9
28/13	54.5 ± 2.6	1277 ± 162	23.4 ± 7.9
35/20	62.2 ± 2.5	3937 ± 288	54.5 ± 13
48/33	86.8 ± 2.7	4714 ± 375	54.3 ± 10
60/45	87.9 ± 3.1	4731 ± 291	53.8 ± 9.6
85/65	88.2 ± 4.0	4683 ± 214	53.1 ± 8.3
120/100	86.6 ± 3.9	4614 ± 183	53.3 ± 7.8
240/120	86.8 ± 2.6	4583 ± 101	52.8 ± 8.1
320/200	85.6 ± 3.1	4566 ± 116	53.3 ± 9.4

Note: ecological niches—liver, pancreas, and gall bladder.

**Table 2:** Dynamics of the infestation intensity and egg production of *O. felineus* in carcinogenesis (experiment).

Experiment period	SO		SO+liver cancer		SO+pancreas cancer		SO+GIST	
	No. of opisthorchii in ecological niches (*M* ± *m*)	Number of eggs per parasite (*M* ± *m*)	No. of opisthorchii in ecological niches (*M* ± *m*)	Number of eggs per parasite (*M* ± *m*)	No. of opisthorchii in ecological niches (*M* ± *m*)	Number of eggs per parasite (*M* ± *m*)	No. of opisthorchii in ecological niches (*M* ± *m*)	Number of eggs per parasite (*M* ± *m*)
85/65	88.2 ± 4.0	53.1 ± 8.3	62.2 ± 2.8^∗^	62.5 ± 3.1	76.4 ± 4.3^∗^	55.3 ± 6.2	79.1 ± 8.1^∗^	57.2 ± 7.8
120/100	86.6 ± 3.9	53.3 ± 7.8	56.3 ± 3.2^∗^	65.4 ± 4.8	72.8 ± 4.0^∗^	61.0 ± 4.2	78.3 ± 4.8^∗^	56.2 ± 2.7
240/120	86.8 ± 2.6	52.8 ± 8.1	48.4 ± 4.1	73.7 ± 4.9^∗^	68.7 ± 4.6	59.3 ± 3.9^∗^	77.8 ± 3.17	56.1 ± 1.9^∗^
320/200	85.6 ± 3.1	53.3 ± 9.4	46.7 ± 3.7	60.32 ± 3.7^∗^	62.4 ± 4.1	68.6 ± 2.4^∗^	75.2 ± 2.9	56.7 ± 1.8^∗^

Note: ^∗^Statistically significant differences in comparison with the SO group (*p* < 0.05).

## Data Availability

All data underlying the findings described in the manuscript are fully available without restriction. All relevant data are within the manuscript.
